# Occupational identity, job satisfaction and their effects on turnover intention among Chinese Paediatricians: a cross-sectional study

**DOI:** 10.1186/s12913-020-05991-z

**Published:** 2021-01-04

**Authors:** Wanjun Deng, Zhichun Feng, Xinying Yao, Tingting Yang, Jun Jiang, Bin Wang, Lan Lin, Wenhao Zhong, Oudong Xia

**Affiliations:** 1 School of Health Management, Southern Medical University, Guangzhou, 510515 China; 2 BaYi Children’s Hospital, The Seventh Medical Center of PLA General Hospital, Beijing, 100000 China; 3 Zhujiang Hospital, Southern Medical University, Guangzhou, 510280 China

**Keywords:** Paediatricians, Occupational identity, Job satisfaction, Turnover intention

## Abstract

**Background:**

This study contributes to research on the paediatrician shortage by examining occupational identity, job satisfaction and their effects on turnover intention among paediatricians in China.

**Methods:**

A multi-stage stratified random sampling method was employed to conduct a questionnaire survey. Of the 4906 survey recipients, valid data were collected from 4198 of the respondents (85.6%). The participants were from seven geographic regions of China (south, central, north, east, northwest, southwest, and northeast). Paediatricians who volunteered and provided written informed consent participated. All variables including basic socio-demographics and work-related characteristics, occupational identity, job satisfaction and turnover intention were based on available literature, and measured on a 5- point Likert scale. Statistical methods such as exploratory factor analysis (EFA), descriptive analysis, common method bias, one-way ANOVA test, Pearson correlation analysis and mediation analysis were used.

**Results:**

Significant differences were observed among the respondents in terms of turnover intention based on age, education level, marital status, region, the type and grade of practice setting, professional title, years in practise, workload, rest days, and monthly income. Occupational identity and job satisfaction were both negatively related to turnover intention, and occupational identity was positively correlated with job satisfaction (r_1_ = − 0.601, *p* < 0.01; r_2_ = − 0.605, *p* < 0.01). The results also showed that job satisfaction played a mediating role in the association between occupational identity and turnover intention among Chinese paediatricians.

**Conclusions:**

Work conditions, workload and salary are crucial factors of turnover intention among paediatricians in China. Therefore, we suggest that healthcare managers should increase investment in paediatrics, implement salary reforms and dedicate more attention to female and young paediatricians, thus reducing turnover intention among Chinese paediatricians.

## Background

Since China’s health system reform, its infant mortality has decreased from 50.2‰ in 1991 to 6.1‰ in 2018; meanwhile, other child health indicators have improved considerably [1]. Despite steady improvement in paediatric services, China’s child health system is in danger of collapse due to a shortage of paediatricians. As noted by the National Bureau of Statistics of China, the ratio of registered paediatricians aged less than 65 per 1000 children was 0.63 in 2017 [2]. Another survey addressing the characteristics and workload of paediatricians in China described that the distribution of paediatricians was extremely skewed (Gini coefficient 0.61), and that, in 2016, paediatricians were burdened with a greater workload [3]. At the same time, the universal two-child policy implemented on January 1, 2016, which aims to address the country’s population aging trend, has exacerbated the shortage of paediatricians in China [4–7]. Moreover, paediatricians have one of the highest turnover rates among physician specialists [7]. According to the *China Pediatric Resources White Paper*, a total of 14,310 paediatricians, accounting for 10.7% of paediatricians in China, left their position for other professions between 2011 and 2014 [8]. The shortage of paediatricians and a high turnover rate have created challenges for China’s paediatric health care system.

According to Erikson, occupational identity (OI) exists in early growth, adolescence and adulthood, and it can be strengthened and developed constantly throughout the lifespan [9]. Inspired by the formulations of Erikson, Holland conceptualized OI as a stable state with behaviours formed by individuals for their career goals, interests and abilities [10]. In contrast, Savickas proposed the self-concept of changing OI [11], and Vondracek described OI as a dynamic organization of career self-perception [12]. Hence, we assumed that OI refers to the development of self-consciousness and self-identity. Meeus discovered that the achievement and social support of adolescents in the educational environment promote the development of their OIs [13]. Early research explored OI among healthcare workers. Subsequently, in an investigation of the correlation between nurses’ OI and environment, Leufgen also discovered that the environment has a positive effect on OI [14]. Wu [15] and Zhao [16] revealed that OI was associated with education, the type of practice setting and salary among healthcare workers in China. However, a study conducted by Selma et al. [17] demonstrated that OI was correlated with job satisfaction and an intention to leave the profession among nurses in Turkey. Meanwhile, Zhang et al. [18] confirmed that OI was a strong predictor of turnover intention (TI) among township health inspectors in China.

Job satisfaction has been a topic of research for decades. Hoppock proposed the concept of job satisfaction from psychological and physiological perspectives, and pointed out that job satisfaction refers to a subjective feeling that an employee holds towards his or her job [19]. Locke further recognized job satisfaction as a positive emotional state resulting from evaluations of one’s work or work experience [20]. Job satisfaction is the extent to which people like (satisfaction) or dislike (dissatisfaction) their jobs [21], and refers to an attitude or emotional response to one’s tasks as well as to the physical and social conditions of the workplace [22]. In fact, job satisfaction is related to many factors. Manojlovich [23] verified that factors in the practice environment contributed directly to nurses’ job satisfaction. Healy et al. [24] and Ozyurt et al. [25] revealed that job satisfaction is inversely correlated with burnout. Jackson et al. [26] showed that women and young doctors may have a higher risk of dissatisfaction at work. In a study among Australian nurses’ regarding job satisfaction and retention, Cowin and Leanne [27] also confirmed that experienced nurses had relatively stable job satisfaction, and that salary was a significant element of young nurses’ job satisfaction. Additionally, job satisfaction is usually regarded as the most representative antecedent variable to predict the TI of health care providers. A previous study conducted by Porter and Sters [28] identified a significant negative correlation between job satisfaction and TI. Recently, a study among doctors in the district public-private mixed health system of Bangladesh also confirmed a significant negative correlation between job satisfaction and TI [29]. Tao et al. [30] demonstrated that job satisfaction was a strong predicator of TI among paediatricians in China. Furthermore, job satisfaction is often the mediator between other factors and turnover intention. Einar M. Skaalvik [31] explored the relationship between school environment variables and teachers’ sense of belonging, and the results showed that job satisfaction was the intermediary variable between wages and job transfer intention. In accordance with this previous study, Chan SHJ verified [32] the mediating effect of job satisfaction on career adaptability and turnover intention. One result of the study that was consistent with the previous study was that job satisfaction has a negative impact on TI, and that the indirect effect is significant.

Turnover intention (TI), according to Mobley [33], is considered to be the best and intuitive predictor of actual turnover behavior, and it refers to the probability that an employee will voluntarily leave his or her job in the period ahead [34]. Some related research on TI has been undertaken extensively by scholars in various fields. As a study initiated by Mobley [33] pointed out, TI is a summative factor of other turnover-related factors and is significantly related to employee turnover. In another study, Shader [35] proposed that job and role stress are important factors leading to TI. Han et al. [36] indicated that factors such as job burnout, work pressure, workplace, working hours, self-efficacy are predictors of TI. Based on these studies, Scanlan, J. N [37] further explored the factors influencing occupational therapists’ job well-being and TI; he discovered that all indicators of job happiness were significantly related to TI. As recognition has increased regarding the importance of the association between TI and the actual level of organization management [38], a large number of studies have focused on TI in occupational populations. A study among Jordanian nurses in psychiatric units by Alsaraireh et al. [39] found a significant negative correlation between job satisfaction and TI. Zhang et al. [18] demonstrated that professional identity, job satisfaction and work engagement were strong predicators of TI among township health inspectors in China.

**Fig. 1:**
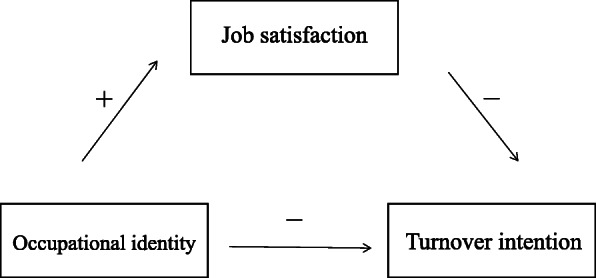
The theoretical model and hypotheses

Based on the above-mentioned theoretical analysis and empirical demonstrations, we attempted to link the relationship and moderating effect among OI, job satisfaction and TI. The model is presented in Table 1 and Fig. 1. We assumed that OI and job satisfaction directly affect TI. Meanwhile, through job satisfaction, OI has an indirect effect on TI. Thus, this study aimed to verify the direct impacts of OI and job satisfaction, and to explore and quantify the influencing mechanism of job satisfaction as an intermediate variable. In a stressful practice environment, paediatricians are subjected to higher workloads and work pressure and relatively low salaries, which may place pediatrician at risk for emotional exhaustion and increased levels of TI [40]. Related to this issue is the benign professional attitude construction of paediatricians. An increased understanding of the complex interrelationship between OI, job satisfaction and TI, and the mechanism of these three, can help to facilitate appropriate measures to counteract the issue of high turnover; this issue deserves more attention.

**Table 1:** The theoretical hypotheses

Hypotheses	
1. Paediatricians’ occupational identity has a direct negative effect on turnover intention	
2. Paediatricians’ job satisfaction has a negative effect on turnover intention	
3. Paediatricians’ occupational identity has an indirect negative effect on turnover intention through the mediating effect of job satisfaction

In recent years, the shortage of paediatricians in China has received extensive attention from all walks of life. On the one hand, the demand for pediatric medical care in China is increasing rapidly, on the other hand, the supply of pediatric medical resources is insufficient. However, what this issue essentially reveals is the bad working condition of the Chinese paediatrician group, which in the study area is not studied before so this study will try to fill the information gap on the working condition of the Chinese paediatrician group. Also we administered a survey to registered paediatricians in China with the aims of identifying key factors that influence how paediatricians perceive their work, exploring the relations between OI, job satisfaction and TI, and developing new and better strategies to address the shortage of paediatricians.

## Methods

### Setting and participants

This study is part of the research project funded by the Humanities and Social Sciences of the Ministry of Education of China, “Exploration on Reasons and Countermeasures for the Shortage of paediatricians in China”. Judging from the experience of sample size summarized by Yuan et al. [41], the sampling ratio is less than 1% when the overall scale is greater than 100,000 people. By the end of 2016, the number of paediatricians in China was 114,010, according to national statistics; thus, the minimum sample size is 1141 (1%). For the purpose of data reliability and validity, the estimated sample size for this survey is 4500. The research object for the influencing factors of TI was in-service paediatricians in medical institutions nationwide. Inclusion criteria: 1) A signed labour contract or other related labour agreement with a public medical institution; 2) Registered doctors with a license to practicing doctors; and 3) A position in relevant paediatrics departments of public medical institutions.

From August 2018 to December 2018, this study employed a multi-stage stratified random sampling method to conduct a questionnaire survey. First, according to China’s geographical divisions, we divided the country into 7 regions of South China, Central China, North China, East China, Northwest China, Southwest China, and Northeast China, and according to the proportion of provinces in each region, we randomly selected 4 provinces in East China, 2 provinces each in South China, North China, Southwest China and Northwest China,and 1 province each in Central China and Northeast China. Second, we randomly selected 3 cities in all the provinces. Then, according to the proportions and grades of local medical institutions, we randomly selected 10 medical institutions and then randomly selected 10 to 12 paediatricians (including attending physicians and residents) from each institution.

Data were collected through self-administered questionnaires based on collaboration with the Pediatric Society of the Chinese Medical Doctor Association (PS-CMDA) and the Neonatal Society of the Chinese Medical Doctor Association (NS-CMDA), which included four parts in addition to the cover letters. To reduce survey bias, this study adopted a self-administered questionnaire survey method. The cover letter informed the respondents that their information was confidential and used only for academic research. Every doctor who accepted the questionnaire survey voluntarily completed the questionnaire after providing informed consent and participated in the questionnaire survey. Secondly, the project team conducted standardized training for multiple investigators, including the research purpose, investigation content, investigation methods, and questionnaire distribution, recovery, and entry. Due to the long distance for some investigators, the training was conducted online. A total of 4906 questionnaires were distributed, and 4198 questionnaires were actually collected. The questionnaire recovery rate was 85.6%.

### Study measures

The study questionnaire, which contained 46 items, was based on an accepted international scale translated into Chinese. The predicted time to finish the questionnaire was 10–15 min.

- Part 1 included basic socio-demographic and work-related characteristics, including age, gender, education, marital status, region, the type and grade of practice setting, professional title, years in practice, the number of hospital beds, rest days in a week and monthly salary.
- In part 2, OI was measured using a 10-item Chinese version of the OI scale (CPIS) developed by Cai (2003) based on the original Occupational Identity Scale (Tyler & McCallum, 1998) [42, 43]. Items included “My present job makes me feel very proud” and 9 other items. The CPIS has a 5-point response format, ranging from 1 = “strongly disagree” to 5 = “strongly agree”. Total scores can range from 10 to 50 and higher scores indicate higher levels of OI.
- In part 3, job satisfaction was measured using the Chinese version of the Minnesota Satisfaction Questionnaire-Short Version (MSQ-short version) developed by Wu based on the MSQ-short version developed by Weiss [44], a well-known and stable instrument used to measure job satisfaction. The MSQ-short version includes 20 items and two subscales: Intrinsic Job Satisfaction (IJS) (consisting of 12 items, including activity, independence, variety, social status, moral value, security, social service, authority, ability utilization, responsibility, creativity, achievement) and Extrinsic Job Satisfaction (EJS) (consisting of 6 items including supervision-human relation, supervision-technical, company policies and practices, salary, recognition). Two additional items explore satisfaction with working conditions and co-worker relationships. The results are reported as General Job Satisfaction (GJS), which is the total score for all 20 items and subscale scores for intrinsic job satisfaction and extrinsic job satisfaction [45, 46]. The 20 items in the MSQ-short version are rated on a 5-point Likert scale (ranging from 1= “very dissatisfied with this aspect of my job” to 5= “very satisfied with this aspect of my job”). Item responses are summed or averaged to create a total score ranging from 20 to 100, with a lower score corrseponding to a lower the level of job satisfaction [46].
- Part 4 consisted of a 4-item instrument measuring TI developed by Farh that is considered to have high internal uniformity and retest reliability [47, 48]. The responses are based on a 5-point Likert scale (ranging from 1 = “strongly disagree” to 5 = “strongly agree”). Total scores can range from 4 to 20 and higher scores indicate higher levels of TI.

### Statistical analysis

We analysed the collected data with SPSS 23.0 with the following methods and objectives: 1) Exploratory factor analysis (EFA) was used to scientifically assess the responsibility and validity of the whole questionnaire. 2) Descriptive analysis was performed to determine the numbers (N) and percentages (%) for socio-demographic and work-related characteristics as well as the means and standard deviations (SDs) for OI, job satisfaction, and TI. 3) The effects of an unmeasured latent method factor were controlled in a common method bias: three models were constructed with four items for OI (10 items), internal job satisfaction (12 items), external job satisfaction (6 items), and TI (4 items), and model differences were compared in AMOS 24.0; after adding the common method factor to the four-factor model, several indicators including the root mean square error of approximation (RMSEA) and standardized root mean square residual (SRMR) decreased or increased by no more than 0.05, while the comparative fit index (CFI) and Tucker-Lewis index (TLI) decreased or increased by no more than 0.1, which reflecting an acceptable fit between the current data and the hypothesized model. 4) One-way analysis of variance was used to compare group differences in the socio-demographic variables. 5) Pearson correlation analysis was applied to OI, job satisfaction and TI. 6) The bootstrap method was used to analyse the mediating effect: internal job satisfaction and external job satisfaction were set as mediator variables(M), OI was set as the independent variable (X), and TI was set as the outcome variable (Y). Then,model 6 and 5000 bootstrap samples were selected in SPSS PROCESS; the X → Y path regression equation and X/M → Y path regression equation must be significant, and the confidence interval for indirect effects did not include 0, indicating that the effect of mediation was statistically significant.

### Reliability and validity

In accordance with the EFA results, the Kaiser-Meyer-Olkin (KMO) value of the CPIS was 0.924, and Bartlett’s test of sphericity was significant (*χ*^*2*^ = 21,661.103, *P* < 0.01). The KMO of the MSQ was 0.941, and Bartlett’s test of sphericity was significant (*χ*^*2*^ = 35,957.862, *P* < 0.01). The Kaiser-Meyer-Olkin (KMO) of TI was 0.784 and bartlett’s test of sphericity was significant (*χ*^*2*^ = 5533.583, *P* < 0.01). All KMO values were greater than 0.70, indicating a better possibility for factor analysis. Reliability analysis was performed and we found that Cronbach’s α values for CPIS, MSQ and TI were 0.905, 0.919 and 0.810, respectively. A coefficient above 0.8 generally indicates acceptable reliability of a scale.

## Results

### Descriptive analysis of the socio-demographic and worked-related characteristics

A total of 4906 paediatricians were invited to participate in the study, and valid data were collected from 4198 of the respondents (85.6%). Table 2 provides the descriptive statistics for the individual variables analysed in this study. The age of the respondents varied between 20 and 74 years, and approximately 85% of the respondents were younger than age 50 years of age. Most of the participants were female (70.1%), and the most paediatricians in China are female. In terms of education, most of the respondents had a university degree or above (94.7%), and most held a bachelor’s degree. Most of the participants were married at the time of the study (88.7%).

**Table 2:** Descriptive data for the socio-demographic and worked-related characteristics of paediatricians

Characteristic	Total sample	
	N	%
**Age (years)**		
20–29	594	14.1
30–39	1635	38.9
40–49	1337	31.8
50–59	610	14.5
> = 60	22	0.05
**Gender**		
Male	1256	29.9
Female	2942	70.1
**Education**		
College graduate	223	5.3
Bachelor’s degree	3035	72.3
Master’s degree	817	19.5
Doctorate degree	123	2.9
**Marital**		
Not married (single and others)	475	11.3
Married	3723	88.7
**Region**		
Northeast China	534	12.7
North China	537	12.8
East China	632	15.1
South China	659	15.7
Central China	597	14.2
Northwest China	635	15.1
Southwest China	604	14.4
**Type of practice setting**		
General hospital	2980	71.0
Children’s hospital	342	8.1
Maternal and child care service centre	806	19.2
Other	70	1.7
**Grade of practice setting**		
Primary	100	2.4
Secondary	1700	40.5
Tertiary	2342	55.8
Not rated	56	1.3
**Professional title**		
Junior	1231	29.3
Middle	1286	30.6
Senior	1681	40.0
**Years in practice**		
< 1 year	156	3.7
1–5 years	894	21.3
6–10 years	868	20.7
11–15 years	606	14.4
16–20 year	459	10.9
> 20 years	1215	28.9
**Number of hospital beds**		
< 5	379	9
5–10	1634	38.9
11–15	1243	29.6
16–20	409	9.7
> 20	533	12.7
**Rest day / week**		
0 day	1568	37.4
1 day	1790	42.6
2 days	821	19.6
3 days	19	0.5
**Monthly income (CNY)**		
< 10,000	3462	82.5
10,001–20,000	637	15.2
20,001–30,000	73	1.7
> 30,000	26	0.6

With respect to practice settings, most of the participants worked in a government general hospital (71%), and smaller percentages worked in a children’s hospital (8.1%) or other settings (1.7%). A considerable proportion of the respondents worked in a secondary (40.5%) or tertiary (55.8%) practice setting. Among the respondents, 29.3% were junior doctors, 30.6% were middle doctors, and 40.0% were senior doctors. More than half of the participants reported working for over 10 years (54.2%). Most of the respondents reported being responsible for 5–15 hospital beds (68.5%) and were most likely to have 1 day of rest each week (42.6%).A monthly salary < 10,000 yuan was reported by 82.5% of the respondents.

### Common method bias analysis of occupational identity, job satisfaction and turnover intention

We controlled for the effects of an unmeasured latent method factor to verify whether a common method bias existed. The single-factor model had a poor fit, and the four-factor model had a good fit (Table 3). After adding the common method factor to the four-factor model, the fitting index of model was not substantially improved. The RMSEA and SRMR reductions were not more than 0.05 (Table 3), and the CFI and TLI increases were not greater than 0.1 (Table 3), indicating that the fit of the model with the method factor did not result in significant improvement, and that although a common method bias may exist, it had little impact on the study [49].

**Table 3:** Common method bias

Model	χ2	df	RMSEA	SRMR	TLI	CFI
Single factor model	19,055.63	464	0.098	0.071	0.708	0.727
Four-factor model	11,181.44	458	0.075	0.057	0.829	0.842
Four-factor model + Common method factor	8802.427	429	0.068	0.051	0.858	0.877

### The status quo of occupational identity, job satisfaction and turnover intention among Paediatricians

The mean score for OI was 28.52, with scores ranging from 10 to 50 (SD = 8.516) (Table 4). The ANOVA revealed that with the exception of marital status, all the socio-demographic and work-related characteristics were significantly associated with OI (Tables 4 and 5).

**Table 4:** Descriptive data and ANOVA results for the socio-demographic characteristics of paediatricians

Characteristic	Occupational identity		General job satisfaction		Intrinsic job satisfaction		Extrinsic job satisfaction		Turnover intention	
	Mean (SD)	***P***	Mean (SD)	***P***	Mean (SD)	***P***	Mean (SD)	***P***	Mean (SD)	***P***
**Age (years)**		**		**		**		**		**
20–29	28.02 (8.51)		61.64 (13.25)		38.14 (8.10)		17.03 (4.61)		11.94 (3.51)	
30–39	26.81 (8.13)		59.93 (12.31)		37.42 (7.69)		16.13 (4.46)		11.82 (3.21)	
40–49	29.21 (8.51)		61.14 (11.60)		38.80 (7.21)		15.98 (4.38)		11.06 (3.11)	
50–59	31.82 (8.31)		63.85 (12.14)		40.23 (7.64)		16.97 (4.52)		10.20 (3.30)	
> = 60	35.23 (8.11)		64.91 (8.18)		41.36 (4.44)		17.00 (3.51)		9.91 (2.00)	
**Gender**		**		**		**		**		–
Male	27.61 (8.82)		59.64 (13.03)		37.31 (8.25)		15.96 (4.60)		11.48 (3.27)	
Female	28.90 (8.36)		61.80 (11.85)		38.85 (7.27)		16.50 (4.42)		11.30 (3.30)	
**Education**		**		–		–		**		*
College graduate	29.46 (9.39)		61.89 (12.13)		39.34 (7.51)		16.08 (4.54)		11.19 (3.24)	
Bachelor’s degree	28.15 (8.48)		60.91 (12.22)		38.28 (7.60)		16.21 (4.47)		11.44 (3.31)	
Master’s degree	29.20 (8.14)		61.59 (12.11)		38.41 (7.49)		16.74 (4.38)		11.15 (3.22)	
Doctorate degree	31.41 (9.37)		62.94 (14.09)		39.29 (8.67)		17.10 (5.14)		10.71 (3.33)	
**Marital**		–		–		–		**		**
Not married (single and other)	28.28 (8.30)		61.85 (12.79)		38.14 (7.86)		17.23 (4.40)		11.83 (3.47)	
Married	28.55 (8.54)		61.06 (12.18)		38.42 (7.58)		16.22 (4.48)		11.29 (3.26)	
**Total**	28.52 (8.52)		61.15 (12.25)		38.39 (7.61)		16.34 (4.48)		11.35 (3.29)	

Note: * p < 0.05, ** *p *< 0.01

In the terms of job satisfaction, the distribution of the paediatricians’ responses to the 20 5-point indicators within the 4 dimensions is shown in Tables 4 and 5. The mean total score for general job satisfaction was 61.15, with scores ranging from 20 to 100 (SD = 12.252) (Table 4). In addition, the mean score for intrinsic job satisfaction was 38.39 (ranged from 12 to 60), while the mean score for extrinsic job satisfaction was 16.34 (ranged from 6 to 30). The results of the ANOVAs revealed that age, gender, region, the grade of practice setting, professional title, years in practice, workload, rest days, and monthly income were significantly associated with general job satisfaction (Tables 4 and 5).

**Table 5:** Descriptive data and ANOVA results for the work-related characteristics of paediatricians

Characteristic	Occupational identity		General job satisfaction		Intrinsic job satisfaction		Extrinsic job satisfaction		Turnover intention	
	Mean (SD)	***P***	Mean (SD)	***P***	Mean (SD)	***P***	Mean (SD)	***P***	Mean (SD)	***P***
**Region**		**		**		**		**		**
Northeast China	27.62 (7.68)		61.19 (11.72)		38.52 (7.35)		16.40 (4.34)		11.73 (3.11)	
North China	29.97 (8.74)		62.12 (13.31)		38.93 (8.13)		16.68 (4.79)		10.95 (3.38)	
East China	29.21 (8.26)		61.14 (12.39)		38.39 (7.57)		16.15 (4.49)		10.92 (3.17)	
South China	28.33 (8.47)		61.77 (11.67)		38.49 (7.43)		16.79 (4.18)		11.36 (3.23)	
Central China	27.95 (8.52)		60.60 (11.58)		38.08 (7.39)		16.07 (4.27)		11.58 (3.09)	
Northwest China	27.60 (8.74)		59.03 (12.25)		37.46 (7.53)		15.35 (4.55)		11.64 (3.25)	
Southwest China	29.03 (8.87)		62.39 (12.60)		38.96 (7.79)		16.98 (4.56)		11.29 (3.68)	
**Type of practice setting**		**		–		–		**		**
General hospital	28.22 (8.50)		60.87 (12.15)		38.25 (7.56)		16.19 (4.42)		11.44 (3.30)	
Children’s hospital	29.47 (8.60)		62.55 (12.39)		38.93 (7.46)		17.21 (4.66)		10.70 (3.34)	
Maternal and child care service centre	29.00 (8.46)		61.50 (12.68)		38.56 (7.87)		16.47 (4.64)		11.28 (3.22)	
Other	30.73 (8.62)		62.43 (10.49)		39.71 (7.11)		16.57 (3.62)		11.56 (3.21)	
**Grade of practice setting**		**		**		**		**		**
Primary	28.31 (8.95)		59.46 (11.39)		37.54 (7.45)		15.77 (3.99)		11.49 (3.37)	
Secondary	27.73 (8.49)		60.01 (12.26)		37.93 (7.73)		15.74 (4.47)		11.69 (3.25)	
Tertiary	29.08 (8.45)		62.04 (12.14)		38.73 (7.46)		16.79 (4.45)		11.10 (3.29)	
Not rated	29.39 (9.84)		61.89 (14.90)		39.36 (9.63)		16.23 (4.82)		11.41 (3.39)	
**Professional title**		**		**		**		**		**
Junior	27.70 (8.62)		61.20 (13.04)		37.89 (8.00)		16.85 (4.60)		11.81 (3.44)	
Middle	26.81 (8.11)		59.02 (11.70)		37.25 (7.37)		15.47 (4.32)		11.77 (3.12)	
Senior	30.42 (8.38)		62.75 (11.83)		39.62 (7.32)		16.62 (4.42)		10.70 (3.20)	
**Years in practice**		**		**		**		**		**
< 1 year	31.13 (8.36)		64.91 (13.47)		39.58 (8.08)		18.43 (4.75)		10.92 (3.45)	
1–5 years	27.88 (8.54)		61.12 (12.88)		37.91 (7.90)		16.83 (4.54)		11.68 (3.44)	
6–10 years	26.69 (8.01)		59.93 (12.21)		37.57 (7.64)		15.95 (4.42)		12.00 (3.13)	
11–15 years	27.20 (8.58)		60.25 (12.00)		37.90 (7.45)		15.99 (4.38)		11.61 (3.20)	
16–20 year	28.23 (8.36)		59.92 (11.83)		37.86 (7.47)		15.66 (4.42)		11.24 (3.18)	
> 20 years	30.72 (8.37)		62.49 (11.71)		39.61 (7.28)		16.41 (4.41)		10.61 (3.21)	
**Number of hospital beds**		**		**		**		**		**
< 5	30.03 (8.51)		63.02 (13.08)		39.36 (7.90)		17.16 (4.77)		10.63 (3.36)	
5–10	28.38 (8.15)		61.46 (11.87)		38.62 (7.30)		16.39 (4.38)		11.45 (3.16)	
11–15	27.75 (8.42)		60.36 (11.78)		37.82 (7.37)		16.15 (4.38)		11.58 (3.28)	
16–20	27.90 (8.70)		59.64 (12.74)		37.46 (8.13)		15.85 (4.44)		11.40 (3.35)	
> 20	30.14 (9.34)		61.89 (13.24)		39.04 (8.26)		16.39 (4.76)		11.00 (3.49)	
**Rest days / week**		**		**		**		**		**
0 day	26.29 (8.43)		58.15 (12.29)		36.54 (7.74)		15.41 (4.41)		12.16 (3.22)	
1 day	29.32 (8.38)		62.27 (12.02)		39.12 (7.43)		16.63 (4.45)		11.01 (3.20)	
2 days	30.90 (7.98)		64.32 (11.43)		40.25 (6.98)		17.41 (4.35)		10.55 (3.28)	
3 days	34.53 (6.35)		66.89 (12.17)		41.68 (7.08)		18.11 (4.78)		10.89 (3.41)	
**Monthly income (CNY)**		**		**		**		**		**
< 10,000	27.86 (8.46)		60.42 (12.22)		37.96 (7.60)		16.08 (4.47)		11.61 (3.24)	
10,001–20,000	31.19 (8.02)		63.95 (11.68)		40.01 (7.19)		17.31 (4.25)		10.21 (3.19)	
20,001–30,000	33.03 (8.05)		68.34 (10.74)		42.42 (6.35)		19.07 (4.22)		9.19 (3.32)	
> 30,000	37.46 (8.12)		70.77 (14.87)		44.42 (9.94)		19.19 (5.36)		10.54 (4.07)	

Note: * p < 0.05, ** *p* < 0.01

The mean total score for TI was 11.35, with scores ranging from 4 to 20 (SD = 3.289) (Table 4). The research results also showed that there were significant differences among the respondents in terms of TI based on age, education, marital status, region, the type and grade of practice setting, professional title, years in practise, workload, rest days, and monthly income (Tables 4 and 5).

### Pearson correlation analysis of occupational identity, job satisfaction and turnover intention

Correlation analysis was conducted on the OI, job satisfaction and TI among Chinese paediatricians (Table 6). The study validated that the level of OI was positively correlated with the respondents’ scores for general job satisfaction as well as intrinsic job satisfaction and extrinsic job satisfaction (r_1_ = 0.702, *p* < 0.01; r_2_ = 0.704, *p* < 0.01; r_3_ = 0.564, *p* < 0.01, respectively). The study validated that the level of TI was negatively correlated with the respondents’ scores for OI, general job satisfaction, intrinsic job satisfaction and extrinsic job satisfaction (r_1_ = − 0.601, *p* < 0.01; r_2_ = − 0.605, *p* < 0.01; r_3_ = − 0.563, *p* < 0.01; r_4_ = − 0.557, *p* < 0.01, respectively).

**Table 6:** Pearson bivariate correlations

Item	Mean	SD	1	2	3	4
1) Occupational identity	28.52	8.516	NA			
2) General job satisfaction	61.15	12.252	.702**	NA		
3) Intrinsic job satisfaction	38.39	7.607	.704**	.953**	NA	
4) Extrinsic job satisfaction	16.34	4.481	.564**	.878**	.706**	NA
5) Turnover intention	11.35	3.289	−.601**	−.605**	−.563**	−.557**

Note: **Correlation is significant at the *P* < 0.05 level, *Correlation is significant at the *P *< 0.01 level

### Mediating effect analysis of occupational identity, job satisfaction and turnover intention

**Fig. 2:**
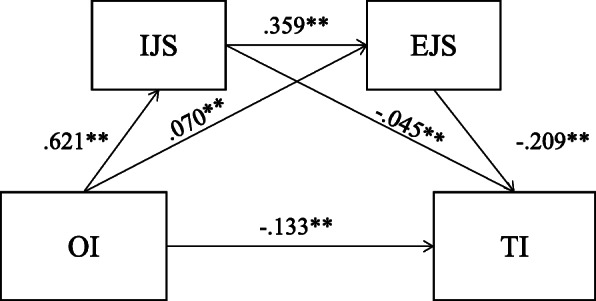
Mediating effect modelNote: **Correlation is significant at the P < 0.05 level

We used the bootstrap method to analyse the mediating effect, which showed that intrinsic job satisfaction and extrinsic job satisfaction mediate relationships between OI and TI. The direct effect refers to X → Y path regression, and the indirect effect refers to X/M → Y path regression. The total effect is the sum of the direct effect and the indirect effect, and the relative mediating effect is the proportion of the indirect effect in the total effect. Thus, the total indirect effect was 0.09, and the total effect was 0.223. We also found that OI to EJS was 0.070 and IJS to TI was − 0.045. They were far less than OI to IJS (0.621), IJS to EJS (0.359) and EJS to TI(− 0.209). So we used a serial mediation model to explain the mediating effect of OI, IJS, EJS and TI. To some extent, the mediating effect of OI to TI through IJS and then EJS were far more than themselves. The mediating effect analysis revealed that OI generates TI through four paths: First, OI directly affects TI(X → Y), and the direct effect was − 0.133 (−0.145, −0.120) (Fig. 2). Second, OI indirectly affects TI through intrinsic job satisfaction(X → M1 → Y). The indirect effect was − 0.028 (−0.039, −0.017), and the ratio in the mediating effect was 12.56% (Table 7). Third, OI indirectly affects TI through extrinsic job satisfaction(X → M2 → Y). The indirect effect was − 0.015 (−0.019, −0.011) and the ratio in the mediating effect was 6.73% (Table 7). Fourth, OI indirectly affects TI through intrinsic job satisfaction and extrinsic job satisfaction(X → M1 → M2 → Y). The indirect effect was − 0.047 (− 0.053, − 0.040), and the ratio in the mediating effect was 21.08% (Table 7).

**Table 7:** Mediating effects analysis of occupational identity on turnover intention

Mediation variable	Indirect effect	Boot SE	Boot CI		Relative mediating effect/%
			Lower bound	Upper bound	
IJS	−.028	.006	−.039	−.017	12.56
EJS	−.015	.002	−.019	−.011	6.73
IJS EJS	−.047	.003	−.053	−.040	21.08

## Discussion

The study explored the effects of OI, job satisfaction and TI among paediatricians in China. Our findings demonstrate that OI, job satisfaction, and TI are prevalent among Chinese paediatricians. Furthermore, we show that paediatricians with increased job satisfaction and OI were significantly less likely to have a strong TI.

Of the 12 intrinsic job satisfaction items, workload received the lowest score. According to data provided by the PS-CMDA, in Beijing Children’s Hospital, each paediatrician should offer 80 to 100 visits each day and sometimes up to 150 visits per day. Similar conditions have also been observed in other paediatric medical centres [50]. Additionally, the number of hospital beds and rest days are two of the major factors affecting paediatricians’ TI. On the whole, compared to the participants responsible for≦ 10 hospital beds, paediatricians responsible for> 10 hospital beds had lower OI and job satisfaction results and higher TIs. According to Wang (2010), the standard number of hospital beds for which paeditricians are responsible in tertiary general hospitals is 9.11 [51]. In our study, 52.0% of the paediatricians were responsible for > 10 hospital beds, and more than half of the participants were responsible for more hospital beds than the number recommended in related reference criteria. In addition, regarding the rest days, 80.0% of the participants took 0 or only 1 rest day per week (Table 2). On the one hand, with the implementation of the two-child policy, the scope of the new baby boom has increased demands for children’s health services and necessitated more talented practitioners in paediatrics. On the other hand, paediatrics departments are subjected to the phenomenon of turnover among paediatricians. The departure of paediatricians with rich experience, especially young paediatricians, is detrimental for medical institutions. According to the China Pediatric Resources White Paper, from 2011 to 2014, the loss rate of paediatricians under 35-years of age was 14.6%, the loss rate of those aged 35-to-45-years was 11%, and the loss rate of those aged 45-to-60-years was 6.8% [8]. Therefore, the heavy workload is a product of the shortage of talent and the increasing demand for services.

With a limited paediatric workforce and increasing demands for child health care, paediatricians are facing unprecedented challenges. Of the 6 extrinsic job satisfaction items, salary received the lowest score. At the same time, salary is one of the significant direct and indirect predictors of TI. Salary has been identified as a contributing factor to a high level of TI [52–54]. A survey performed by the PS-CMDA found that approximately 96% of paediatricians were not satisfied with their salaries in 2011 [50]. The average salary of China’s urban employees in the financial sector reached 122,851 CNY in 2017, according to national data, and that of urban employees in the computer services and software industry was 133,150 CNY; by comparison, the salary of paediatricians lagged behind those of the two other industries [55]. On the one hand, the low salary of physicians is strictly controlled by the government to maintain affordable health care [56]. According to a survey conducted by the PS-CMDA, in 2015, the workload of paediatricians in general hospitals was 1.68-times that of non-paediatricians, while the income earned by paediatricians was 46% of the income earned by non-paediatricians [57]. Compared to other physicians, because of the limited revenue generated by paediatric departments, an income gap exists, increasing the likelihood of paediatricians experiencing fatigue and frustration.

Through Pearson correlation analysis, we observed active correlations between OI, intrinsic job satisfaction and extrinsic job satisfaction, which have negative correlations with TI. These findings are consistent with the negative correlation identified between OI and TI among Chinese nurses [30, 58], and a study exploring job satisfaction and TI among paediatricians reported the negative impacts of age and job satisfaction on TI [30]. Moreover, the mediating effect analysis revealed that in addition to directly affecting TI, OI also affects internal job satisfaction and then external job satisfaction; the highest indirect effect was 21.08%, indicating tha OI generates TI through internal job satisfaction (12.56%), rather than extrinsic job satisfaction (6.73%), and that internal job satisfaction also affects external job satisfaction. Although internal job satisfaction and external job satisfaction function in the same direction, they have inconsistent roles; thus, other variables must affect the effects of the two, suggesting that future research should also fully explore the impacts of the latent variables internal job satisfaction and external job satisfaction.

### Strengths and limitations of this study

To our knowledge, this is the first study to investigate OI, job satisfaction and TI at a national level and to analyse relationships between OI, job satisfaction and TI among paediatricians in China. We invested extensive time and energy in investigating many paediatricians to achieve a representative sample by planning a reasonable method for sample selection in China.

There are several limitations to our study. This is only a cross-sectional study, and no causal inferences can be made. In addition, because the questionnaire involves personal privacy, some respondents may have concealed their real experiences, resulting in a potential reporting bias in the study. Third, the study focused only on the relationships between OI, job satisfaction and TI among paediatricians. The study was conducted from a unilateral view, and some factors may have been ignored. Therefore, we should expand the field of investigation and identify additional problems in future research. On the basis of the research, in-depth interviews with stakeholder groups can be conducted to introduce new variables of research value to explore influencing factors. Through a follow-up survey of paediatricians and cohort research, we can understand the true status and needs of paediatricians while conducting corresponding empirical research on the salaries of paediatricians and the promotion of professional titles.

## Conclusions

Paediatricians play a vital role in the paediatric health system in China. The results show that higher OI and job satisfaction contribute to a decreased TI, providing not only new ideas for explaining the resignation of paediatricians, but also new possible and feasible methods to reduce the tendency towards resignation. Based on the results, we suggest that healthcare managers should improve vacation polices, increase financial investments in paediatric departments, fund projects including paediatric infrastructure, scientific research, and education by providing grant allowances to paediatricians, and most importantly, reform salaries for paediatricians. In addition, the government should focus on the rights of young paediatricians, as they represent a large percentage of Chinese paediatricians but have a high level of TI, provide them with more talent selection and advanced learning opportunities, and strengthen their psychological well-being.

## Data Availability

All authors confirm that the data in this study will be made available from the corresponding author upon reasonable request.

## Abbreviations

OI: Occupational identity
JS: Job satisfaction
TI: Turnover intention
GJS: General job satisfaction
IJS: Intrinsic job satisfaction
EJS: Extrinsic job satisfaction
PS-CMDA: The Pediatric Society of the Chinese Medical Doctor Association
NS-CMDA: The Neonatal Society of the Chinese Medical Doctor Association
